# *Chlorella*-induced thrombocytopenia

**DOI:** 10.1590/1516-3180.2018.0263130918

**Published:** 2018-12-13

**Authors:** Irfan Yavasoglu, Atakan Turgutkaya, Zahit Bolaman

**Affiliations:** I MD. Professor, Division of Hematology, Adnan Menderes University Medical Faculty, Aydın, Turkey.; II MD. Fellow, Division of Hematology, Adnan Menderes University Medical Faculty, Aydın, Turkey.; III MD. Professor, Division of Hematology, Adnan Menderes University Medical Faculty, Aydın, Turkey.

Dear Editor,

Many improvements to alternative medicine have emerged recently. In speaking about the effects of these drugs, it is sometimes impossible to consider their adverse effects adequately.

*Chlorella vulgaris* is a unique single-celled species of freshwater microalga with grass-like odor. It has been used as a form of nutritional support. Clinical trials have suggested that supplementation with *Chlorella vulgaris* can ameliorate hyperlipidemia and hyperglycemia, and protect against oxidative stress, cancer and chronic obstructive pulmonary disease.^1^ Here, we present a case in which thrombocytopenia was developed after use of this product as an anti-aging treatment.

A 49-year-old healthy female patient who was a medical doctor was admitted to our hospital because of thrombocytopenia. She reported that she not had any previous chronic drug use, but that she was taking *Chlorella* in tablet form, at a dose of 1,080 mg/day. Twenty days after starting to take the *Chlorella*, her platelet count was found to be 27,000/mm^3^, while her leukocyte and hemoglobin levels were normal. We learned that a hemogram that had been produced one month earlier (i.e. before she started to use this medicine) was completely normal. The findings from a blood smear were also consistent with thrombocytopenia. Therefore, use of this drug was immediately stopped. Her platelet count then began to improve and became normal after two weeks.

So far, 39 cases of adverse effects from *Chlorella* have been notified to the United States Food and Drug Administration (FDA), and three of them (7.69%) have involved thrombocytopenia. Among these cases, three were males aged over 60 years and all of them had histories of using ornithine and imatinib.^2^ Our case was female and did not have any medical illness.

The adverse effects of alternative products that are made available without their having gone through the various phases of drug trials always need to be considered. Use of these drugs should always be questioned, without exception, if a low platelet count is found.

## INFORMED CONSENT

Informed consent was obtained from the patient who is reported in this study.
